# Revision of the Most Primitive Taxa of the Family Gyrodactylidae (van Beneden et Hesse, 1864) (Platyhelminthes, Monopisthocotyla) Based on ITS rDNA Phylogeny

**DOI:** 10.3390/genes15091236

**Published:** 2024-09-23

**Authors:** Jakub Janulewicz, Maciej Pietkiewicz, Marek S. Ziętara

**Affiliations:** Laboratory of Molecular Evolution and Bioinformatics, Faculty of Biology, University of Gdańsk, Wita Stwosza 59, 80-308 Gdańsk, Polandmaciej.pietkiewicz@phdstud.ug.edu.pl (M.P.)

**Keywords:** Platyhelminthes, ITS rDNA, molecular phylogeny, Gyrodactylidae, *Cichlidarus* gen. nov., *Iraqemembranatus* gen. nov., *Macracanthus* gen. nov., *Rysavyius* gen. nov

## Abstract

Background: For the past 25 years, the ITS rDNA (ITS1-5.8S-ITS2) of Gyrodactylidae has been crucial for species identification, description, and phylogeny. This family includes 25 genera parasitizing marine and freshwater fish, initially distinguished by morphological differences in attachment and/or male copulatory organs. *Gyrodactylus* Nordmann, 1832, the most species-rich genus, has approximately 500 described species and an additional 25,000 species suspected. The genus is not monophyletic, and the functionally adaptive nature of morphological diagnostic characters complicates the delimitation of new genera. Methods: A phylogeny based on ITS rDNA was proposed to address these challenges, using only complete sequences of primitive taxa. Fifty-four sequences were aligned with the MUSCLE v5.1 algorithm, creating a 1590 ps long matrix. Maximum Likelihood (ML) and Bayesian Inference (BI) methods with the models TVM+F+G4 and SYM+G4 for ITS1–ITS2 and 5.8S, respectively, were inferred using IQ-TREE v2.3.5 and BEAST v2.7.6.0. Results: The findings revealed eleven main lineages. Four of them are proposed for classification into new genera: *Cichlidarus* gen. nov., *Iraqemembranatus* gen. nov., *Macracanthus* gen. nov., and *Rysavyius* gen. nov. Elevating the subgenus *G*. (*Gyrodactylus*) to genus rank was supported. Conclusions: The presented phylogeny provides a foundation for developing a classification system within Gyrodactylidae that is both reasonable and comprehensive.

## 1. Introduction

The phylum Platyhelminthes Minot, 1876 occupies a pivotal position in early metazoan evolution. It includes free-living organisms and probably the largest clade of obligate parasites, which is called Neodermata Ehlers, 1985. These are parasites of both invertebrates and vertebrates. Some of them are among the most important medically and economically. Traditionally, they are subdivided into three classes: Monogenea Carus, 1863, Trematoda Rudolphi, 1808, and Cestoda Rudolphi, 1808. The phylum’s members are therefore highly diverse in their morphology, habitat, biogeography, and life history strategies [1,2,3,4,5].

The Gyrodactylidae (Beneden et Hesse, 1864) family is a group of Platyhelminthes, originally classified as viviparous worms possessing a simple posterior attachment organ (haptor) [6]. This family has since been recognized as a distinct taxon within the Monogenea Carus, 1863 class [5] and is conventionally placed in the Monopisthocotylea Odhner, 1912 subclass. Recently, a comprehensive phylogenomic analysis suggested that the Monogenea class is not monophyletic, and it proposed that Monopisthocotylea should be elevated to the rank of class, designated as Monopisthocotyla [7].

The monophyletic status of the Gyrodactylidae family was confirmed by phylogenetic analyses utilizing sequences of nuclear 18S rDNA and mitochondrial *cox2*. These analyses also confirmed the reinstatement of the family Oögyrodactylidae Harris, 1983, which is a sister group to Gyrodactylidae, for oviparous species [8].

Based on morphological criteria, 23 viviparous genera were described within Gyrodactylidae by 2007; however, 5 of these have since been invalidated, namely, *Neogyrodactylus* Baugh, 1957, *Neogyrodactylus* Prudhoe, 1957, *Paragyrodactyloides* Nunez, 1975, *Paragyrodactyloides* Szidat, 1973, and *Micropolyclithrum* Skinner, 1975. The remaining 18 genera were validated based on either morphology [6] or molecular data [8,9,10,11,12,13,14,15,16,17,18,19,20,21,22,23,24,25,26], consisting of *Accesorius* Jara, An et Cone, 1991, *Acanthoplacatus* Ernst, Jones et Whittington, 2001, *Afrogyrodactylus* Paperna, 1968, *Anacanthocotyle* (Kritsky et Fritts, 1977, *Archigyrodactylus* Mizelle et Kritsky, 1967, *Fundulotrema* Kritsky et Thatcher, 1977, *Gyrdicotylus* Vercammen Grandjean, 1960, *Gyrodactyloides* Bychowsky, 1947, *Gyrodactylus* von Nordmann, 1832, *Isancistrum* de Beauchamp, 1912, *Laminiscus* Palsson et Beverly Burton, 1983, *Macrogyrodactylus* Malmberg, 1956, *Mormyrogyrodactylus* Luus-Powell, Mashego et Khalil, 2003, *Metagyrodactylus* (Baugh, 1957), *Paragyrodactylus* Gvosdev et Martechov, 1953), *Polyclithrum* Rogers, 1967, *Scleroductus* Jara et Cone, 1989, and *Swingleus* Rogers, 1969. Since then, seven additional genera have been described, some with the help of DNA data [15,21,23,27,28], namely, *Citharodactylus* Přikrylová, Shinnet Paladini, 2017, *Diechodactylus* Vianna et Boeger, 2008, *Diplogyrodactylus* Přikrylová, Matĕjusová, Musilová, Gelnar et Harris, 2009, *Gyrocerviceanseris* Cone, Abbott, Gilmore et Burtf, 2010, *Ieredactylus* Schelkle, Paladini, Shinn, King, Johnson, Oosterhout, Mohammed et Cable, 2011, *Scutalatus* Vianna, Boeger et Dove, 2007, and *Tresuncinidactylus* Přikrylová, Barson et Shinn, 2021).

All morphological characters used to differentiate the various genera, are based on features of the haptor and the male copulatory organ (MCO), with genetic analysis also revealing further distinctions between the genera [6,16,18,23,26]. However, the genus *Gyrodactylus* (which is the type genus of the family Gyrodactylidae) remains the most problematic in this context. It is the most species-rich genus within the family, encompassing the majority of its species, many of which present particular challenges for accurate species identification [29,30]. By 2004, approximately 409 valid species were known [31], and despite the addition of new species in the two decades since [13,18,32,33,34,35,36,37,38,39,40,41,42,43,44,45,46,47,48,49,50,51,52,53,54,55,56,57,58,59,60,61,62,63,64,65,66,67,68], the list remains incomplete. Given their typically high host-specificity, the expected number of species is thought to exceed 25,000, which is larger than the number of known fish species. Malmberg [69] divided the genus into six subgenera based on the differences in their excretory system (protonephridial system): *G.* (*Gyrodactylus*), *G*. (*Mesonephrotus*), *G*. (*Metanephrotus*), *G*. (*Paranephrotus*), *G*. (*Neonephrotus*), and *G*. (*Limnonephrotus*). He only examined the protonephridial systems from about 75 species—mostly from the northern part of the Palearctic but he classified about 200 known at the time all over the word. The subgenus *G*. (*Gyrodactylus*) was identified as the most primitive, while *G*. (*Limnonephrotus*) was considered the most derived. Additionally, he proposed a further subdivision of the subgenera into species groups based on haptor morphology. Gläser [70] later supported those divisions but elevated subgenera to the rank of genus, while also combining worms with small excretory bladders and lateral flames in the main canals (*G*. (*Mesonephrotus*)) into a single genus (*Postgyrodactylus*) with those lacking the lateral flames in the main canals (*G*. (*Metanephrutus*)). The remaining names of Malmberg’s subgenera were either left unchanged (e.g., *Gyrodactylus*) or modified as follows: *G*. (*Paranephrotus*) was renamed *Mesogyrodactylus*, *G*. (*Neonephrotus*) became *Anguilladactylus*, and *G*. (*Limnonephrotus*) was designated as *Limnogyrodactylus.* Regrettably, these systematic conventions were not widely adopted by the scientific community, due to the labor-intensive nature of the methodology required to examine the protonephridial systems of live worms.

To address the challenges associated with species identification and genetic relationships, molecular markers were developed in the mid-1990s. In 1997, the first sequence of the internal transcribed spacer ribosomal DNA (ITS rDNA) marker was published (GenBank: Z72477.1), consisting of ITS1-5,8S-ITS2 rDNA sequences from *Gyrodactylus salaris* Malmberg, 1957, a major pathogen of Atlantic salmon (*Salmo salar* L.) in Norway. The marker was developed for the practical identification of *Gyrodactylus* species parasitizing salmonid (Salmonidae) species [71]. While this marker cannot always be sequenced accurately, and it is very difficult to produce sequence alignments at the genus level (as detailed in a dedicated review [6]), it has proven invaluable for gyrodactylid species identification and phylogenetic reconstruction, including the testing of Malmberg’s and Gläser’s systematics. The marker has enabled the reconstruction of novel preliminary phylogenies as well as the assessment of species monophyly [72,73]. The most significant finding of this then-novel approach was the observation that the *Gyrodactylus* species can be divided into two main groups based on the ITS1 rDNA marker length. The more primitive taxa have shorter sequences, while the more derived taxa have longer ITS rDNA sequences [9,74]. Matejusová et al. [11] also observed that the excretory system of *Gyrodactylus* lacks the necessary resolution to reveal the existence of distinct subgenera within the genus. Consequently, they proposed the elimination of the subgenera division, while simultaneously endorsing the classification of species into the species groups. Ziętara and Lumme [75] then directed greater attention toward the genetic relationship and evolutionary patterns observed within *Gyrodactylus*. Within species that possess long ITS rDNA sequences, (assigned by Malmberg to the wageneri [69], salaris, or lavareti [76] groups of *G*. (*Limnonephrotus*)) a novel, closely related group was discovered, with a genetic distance of around 6%. Despite the suggestion made by Malmberg, based on haptor morphology [69,76], they excluded *G. gracilihamatus* Malmberg, 1964 and Gyrodactylus *macronychus* Malmberg, 1957 and included *Gyrodactylus lavareti* Malmberg, 1957 and *G. salaris*, forming a new revised wageneri group based on ITS rDNA relationships, while maintaining the same group name. Unfortunately, this resulted in a misunderstanding 20 years later [77], but upon proper analysis and interpretation, it is evident that the wageneri group as defined by Ziętara and Lumme [75] is also monophyletic in this research [77]. A similar approach was subsequently adopted by incorporating 68 species into an ITS rDNA phylogeny [12], in order to critically evaluate Malmberg’s nomenclature [69]. Specifically, the monophyly of the two European freshwater subgenera, *G*. (*Gyrodactylus*) and *G*. (*Limnonephrotus*), was confirmed. A novel lineage of South American species was also identified. The marine rugiensis group (previously classified as *G*. (*Paranephrotus*) by Gläser [78]) now included the only species of *G*. (*Neonephrotus*) and was identified as the sister group to the freshwater *G*. (*Limnonephrotus*). The subgenera *G*. (*Metanephrotus*) and *G*. (*Mesonephrotus*) were found to form a well-supported monophyletic lineage, as previously suggested by Gläser [70]. However, the freshwater lotae group, which had been classified as *G*. (*Paranephrotus*) by Malmberg [69], was also unexpectedly included in this monophyletic lineage, resulting in the *G*. (*Paranephrotus*) subgenus becoming paraphyletic [12]. Subsequent phylogenetic analyses have corroborated the distinctiveness of this lineage within the *Gyrodactylus* [50,63,79,80], and it is currently well established that the genus *Gyrodactylus* is not monophyletic [8,10,11,16,52].

These findings would suggest that the genus *Gyrodactylus* clearly requires subdivision into smaller genera. However, given the considerable number of undescribed species, such a division might be premature. In this study, we illustrate how contemporary phylogenetic techniques can be employed to identify and differentiate monophyletic assemblages that do not necessarily belong within the genus *Gyrodactylus* sensu Malmberg [69]. We then apply these techniques towards the description of new genera. The phylogenetic analyses presented here are based on complete sequences of the most useful ITS rDNA markers within the studied animals. The most primitive gyrodactylid species of the family Gyrodactylidae were preselected based on the shortest complete sequences of the marker. The obtained monophyletic lineages were then used to verify the systematics for this section of the family diversity.

## 2. Materials and Methods

In order to investigate the genetic relationships of the most primitive members of the Gyrodactylidae, complete ITS rDNA sequences were downloaded from GenBank. Complete sequences from other markers for the species of interest are not yet available. All GenBank accession numbers were included together with species names in all figures presenting the resulting phylogenies. These sequences all exhibited characteristic motifs at the 3′ end of the 18S gene (ATCATTA) and at the 5′ end of the 28S gene (CCTGACC). All sequences were aligned using MUSCLE v5.1 [81], with an alignment map prepared using Biopython and the Matplotlib library. The resulting figures were then modified for clarity in Adobe Photoshop v25.9.1. The resulting multiple sequence alignment (MSA) was then used to infer uncorrected and corrected genetic distances as well as phylogenetic relationships. The genetic distances between and within the genera under investigation were calculated. Two analyses were prepared: the p-distances were calculated using MEGA11v11.0.13 [82] software, and likelihood distances were calculated using IQTree2 v2.2.2.6 software [83] for the same evolutionary models as those employed in the Maximum Likelihood (ML) and Bayesian Inference (BI) analyses (see below for more details). The resulting data were processed using Python’s SeaBorn library, with the resulting figures processed for clarity using Adobe Photoshop v25.9.1. The phylogenetic relationships were inferred using ML and BI. The best-fitting evolutionary model was calculated in ModelFinder [84] for three separate partitions: the ITS1, 5.8S, and ITS2 regions. Using the corrected Akaike Information Criterion (AICc) [85], the following best models were selected: TVM+F+G4, SYM+G4, and TVM+F+G4 for ITS1, 5.8S, and ITS2, respectively. The ML tree was inferred using IQ-TREE v2.3.5 [83] based on evolutionary models, described above, with branch support estimated through nonparametric bootstrap (bp) approximation with 1000 replicates. The BI was performed using BEAST v2.7.6.0 [86,87], with the same evolutionary models employed in ML. Four independent Markov Chain Monte Carlo (MCMC) simulations were performed with 10^6^ generations, a sampling frequency of 100, and a 25% burn-in, with branch support estimated though posterior probability (pp) values. The resulting ML and BI trees were visualized in TreeViewer v.2.2.0 [88] and then edited manually for clarity in Adobe Photoshop v25.9.1.

## 3. Results

### 3.1. Characteristics of the ITS rDNA Marker MSA

The final multiple sequence alignment (MSA) contained 53 sequences belonging to the Gyrodactylidae family and a single sequence belonging to the Oögyrodactylidae family (*Aglaiogyrodactylus ctenistus* Kritsky, Vianna et Boeger, 2007), representing the outgroup. The 3′ end of 18S (ATCATTA) and the 5′ end of 28S (CCTGACC) rDNA motifs are characteristic of viviparous gyrodactylids, and they were used to assess the quality of the analyzed sequences. In the outgroup sequence KF767471.1 (*A. ctenistus*), the 3′ end of 18S (ATCACTA) and the 5′ end of 28S (TCTGACC) rDNA exhibited a single transition, either T/C or C/T, in the fifth and first position, respectively. Once the motifs were confirmed, the flanking regions were excised. The majority of the sequences demonstrated concordance with the anticipated motifs. Three sequences—FR850682.1 (*Gyrodactylus alekosi* Přikrylová, Blažek et Vanhove, 2012), FR850679.1 (*Gyrodactylus rysavyi* Ergens, 1973), and KF680221.1 (*Paragyrodactylus variegatus* You, King, Ye et Cone, 2014)—showed aberrant sequences in the 18S rDNA 3′ ends that did not guarantee the proper quality of the following region. However, these sequences were retained for further analysis because other motifs showed the correct sequences. The quality of the 5′ end of 28S rDNA motifs was generally better, although four additional motifs were identified among the downloaded sequences. The CCTGACCTT motif was identified in the EU678357.1 of *Gyrodactylus jennyae* Paetow, Cone, Huyse, McLaughlin et Marcogliese, 2009. The CCTGACTTC motif was identified in the MN759067.1 of *Gyrodactylus shinni* García-Vásquez, Pinacho-Pinacho, Guzmán-Valdivieso, Calixto-Rojas et Rubio-Godoy, 2021. The CCTGACTCA motif was identified in the MT994561.1 of *Gyrodactylus* sp. 5 and the CCCGACCTC motif in the AJ001843.1 of *Gyrdicotylus gallieni* Vercammen Grandjean, 1960. Revision of these new motif sequences revealed that they likely resulted from single-nucleotide errors within the shorter motifs. In some sequences, the motifs were removed prior to submission.

**Figure 1:**
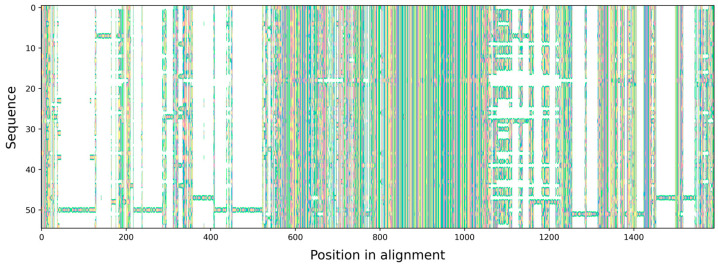
Multiple sequence alignment (MSA) of the ITS rDNA region including all investigated species. Gaps in the sequences are represented as empty spaces. ITS1 rDNA (1–826 ps), 5.8S rDNA (827–986 ps) and ITS2 rDNA (987–1590 ps).

The 5′ end of 5.8S rDNA exhibited a consistent trend of starting with the motif CAACTC. However, in all species of *Macrogyrodactylus* (AJ567672.1, GU252714.1, GU252716.1, GU252717.1, and OM426799.1), one sequence of *Diplogyrodactylus martini* Prikrylová, Matĕjusová, Musilová, Gelnar et Harris, 2009 (AM943008.1), and *G. gallieni* (AJ001843.1), the motif differed from the reference sequence by a single transition (C/T) at the final position, resulting in a CAACTT motif. The sequences AJ249348.1 of *Gyrodactyloides bychowskii* Albova, 1948 and KF767471.1 of *A. ctenistus* exhibited the TAACTC motif, which differed from the expected motifs by a single transition (C/T) at the first position. The 3′ end of 5.8S rDNA GTCGGCT was identical in all species except for KF767471.1 of *A. ctenistus*, which differed by a single transversion at T/A at the last position (GTCGGCA). With the exceptions of AJ249348.1 of *G. bychowskii* (which exhibited a single insertion), AJ567672.1 of *Macrogyrodactylus polypteri* Malmberg, 1956 (which displayed a double insertion), and FR850682.1 of *G. alekosi* (which exhibited a single deletion), all sequences were 157 bp in length. The presence of single-nucleotide indels in these sequences could suggest a lower sequence quality, given the highly conserved nature of the 5.8S rDNA in this group. Only two taxa contained more extensive indels, although these occurred in a region of ITS1 rDNA that is more difficult to align. Once all quality control (QC) steps were completed, the final MSA length was 1590 ps (Figure 1).

The shortest overall sequence identified was AKF767471.1, belonging to the outgroup *A. ctenistus* (Oögyrodactylidae), while the shortest sequence of Gyrodactylidae (AJ001843.1) was that of *G. gallieni* (751 bp). These patterns were consistent with our expectations; however, the longest sequence, KF680221.1 from the species *P. variegatus*, was unexpectedly long, at 1095 bp. Blasting of this sequence revealed that the closest sequence (query cover—46% and per ident—85%) was FR850679.1, belonging to *G. rysavyi*, and this sequence was therefore kept for further analysis. The next-longest sequence, FR850682.1, had a length below 1000 bp and was from *G. alekosi* (926 bp), which was previously identified as being of potentially inferior quality.

### 3.2. Molecular Analysis

#### 3.2.1. Intra- and Interspecific Genetic Distances

The mean genetic distances among species for each genus/subgenus are presented in Table 1. The lowest distance was estimated for species in *Cichlidarus* gen. nov. and *Macrogyrodactylus* (0.09 and 0.1, respectively), and the highest was estimated for species in *Rysavyius* gen. nov. (0.39).

**Figure 2:**
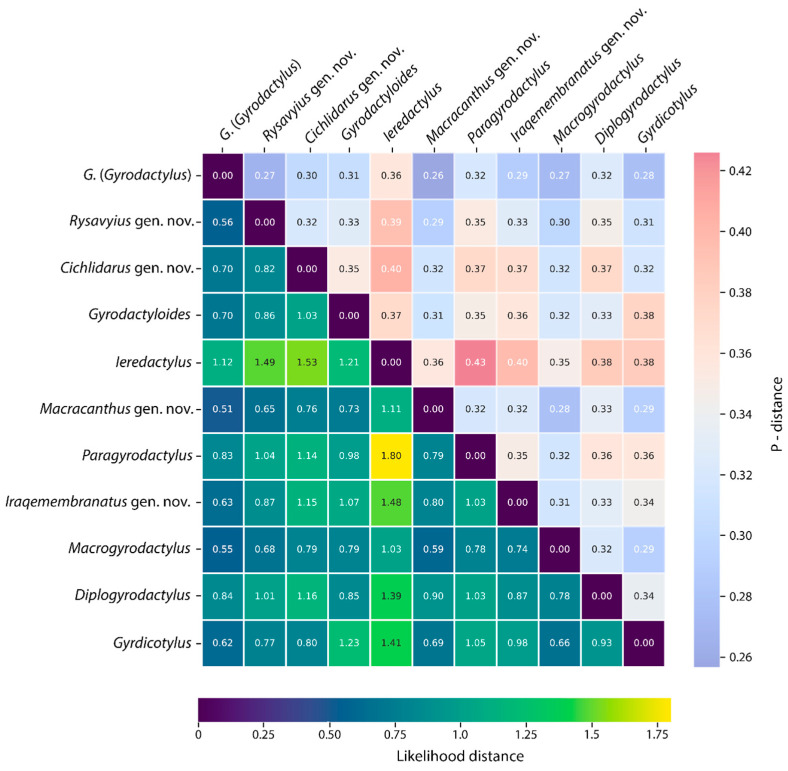
Heatmap displaying pairwise genetic distances among the studied genera/subgenus. The upper right triangle of the matrix represents p-distances calculated in MEGA11 [82], while the lower left triangle shows likelihood distances based on the TVM+F+G4, SYM+G4, and TVM+F+G4 models for the ITS1, 5.8S, and ITS2 regions, respectively, using IQTree2 [83]. The colored bars indicate the range of values for each metric: p-distance (0.26 to 0.42) and likelihood distance (0 to 1.75). The variation in color scales reflects different evolutionary distances between taxa, with warmer colors (yellow/red) denoting more distant relationships and cooler tones (blue/purple) signifying closer relationships.

The mean genetic distances among each genus/subgenus are presented in Figure 2. The lowest genetic distance—0.51—was estimated between *Macrogyrodactylus* and *G*. (*Gyrodactylus*)), while the highest—1.8—was estimated between *Ieredactylus* and *Paragyrodactylus*. It is worth noting that the interspecific genetic distances between the studied taxa are always higher than the intraspecific genetic distances. The genetic distances are distorted by the low number of taxa. *G*. (*Gyrodactylus*) is the only species-rich taxon.

#### 3.2.2. Phylogenic Phylogenetic Inference

**Figure 3:**
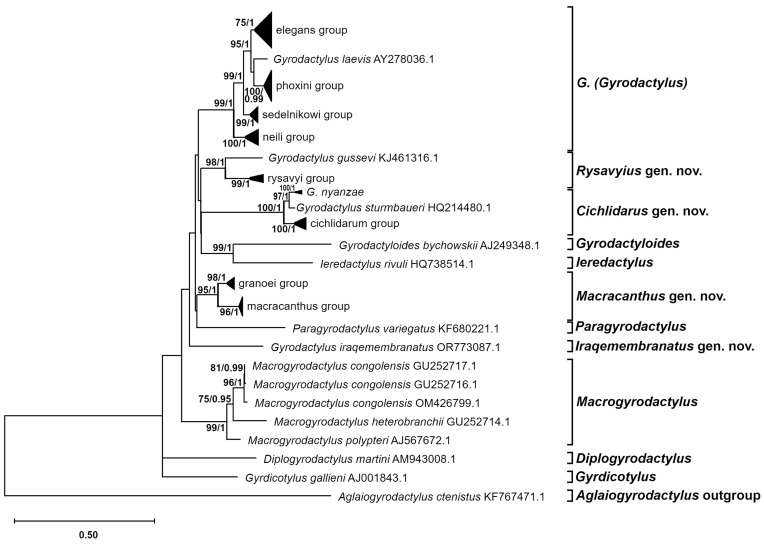
Partially compressed phylogenetic relationships within the Gyrodactylidae family, inferred using ITS rDNA markers, with *Aglaiogyrodactylus ctenistus* (Oögyrodactylidae) as the outgroup. Node numbers represent the ML bootstrap (bp) and BI posterior probability (pp) values. Values below bp 75 and pp 0.95 are not shown. Substitution models used in both analyses: ITS1—TVM+F+G4, 5.8S—SYM+G4, ITS2—TVM+F+G4.

The inferred phylogenetic tree (Figure 3) illustrates the evolutionary relationships between the most primitive taxa within the Gyrodactylidae family. The tree comprises 11 main lineages, 6 of which represent genera of the studied family that were previously described (*Diplogyrodactylus*, *Gyrdicotylus*, *Gyrodactyloides*, *Ieredactylus*, *Macrogyrodactylus*, and *Paragyrodactylus*). *Macrogyrodactylus* is the only genus represented by three species, with a total of five sequences, and its position in the tree is well supported (bp = 99 and pp = 1). The positions of the remaining genera are not inferred with strong support, with the exception of *G. bychowskii* (AJ249348.1) and *Ieredactylus rivuli* Schelkle, Paladini, Shinn, King, Johnson, Oosterhout, Mohammed et Cable, 2011 (HQ738514.1), which are grouped together (bp = 99 and pp = 1).

The other five lineages (out of the eleven mentioned earlier) are represented by species of *Gyrodactylus*, and they are not grouped together in either the ML or BI phylogenies but are positioned within the broader context of other *Gyrodactylidae* genera. One lineage is represented by a single sequence, OR773087.1, derived from *Gyrodactylus iraqemembranatus* Benovics, Rahmouni, Řehulková, Nejat et Šimková, 2024, with the remaining four lineages comprising a larger number of sequences that are grouped together in well-supported lineages. One of the lineages is composed of *Gyrodactylus* sequences, which are grouped with *Gyrodactylus* species assigned to the subgenus *G*. (*Gyrodactylus*). In light of this, we propose the establishment of four new genera to accommodate these newly identified lineages. The new proposed genera are as follows: *Cichlidarus* gen. nov., *Iraqemembranatus* gen. nov., *Macracanthus* gen. nov., and *Rysavyius* gen. nov. Three of these new genera contain multiple species each and are strongly supported by the phylogenetic evidence presented in this study: the new genus *Cichlidarus* gen. nov., with a bootstrap value of 100 and a posterior probability of 1; the new genus *Macracanthus* gen. nov., with a bootstrap value of 95 and a posterior probability of 1; and the new genus *Rysavyius* gen. nov., with a bootstrap value of 98 and a posterior probability of 1.

**Figure 4:**
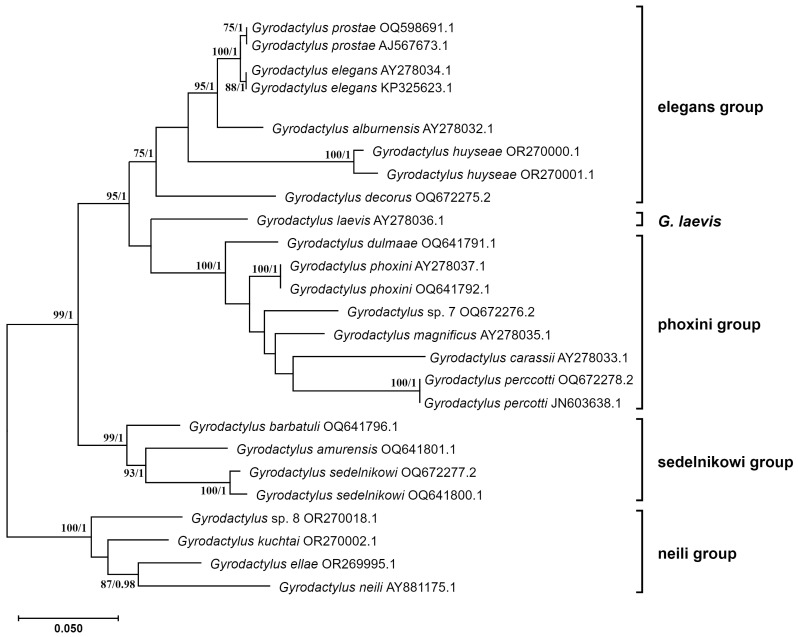
Expanded subtree of *Gyrodactylus* (*Gyrodactylus*) species from phylogenetic relationships inferred using ITS rDNA markers (Figure 3). Node numbers represent the ML bootstrap (bp) and BI posterior probability (pp) values. Values below bp = 75 and pp = 0.95 are not shown. Substitution models used in both analyses: ITS1—TVM+F+G4, 5.8S—SYM+G4, ITS2—TVM+F+G4.

An expanded subtree representing only the *G*. (*Gyrodactylus*) subgenus is presented in Figure 4. The subgenus is clearly composed of five distinct lineages, four of which form species groups. The singleton lineage, *Gyrodactylus laevis* Malmberg, 1957 (AY278036.1), is grouped together with the elegans and phoxini species groups (bp = 95 and pp = 1). The four species groups are well supported: the neili group with bp = 100 and pp = 1, the phoxini group with bp = 100 and pp = 1, and the sedelnikowi group with bp = 99 and pp = 1; despite a lower bp = 75 value for the elegans group, pp = 1 remains high. It is noteworthy that the Eurasian and North American evolutionary lines form sister group relationships with strong support (bp = 99 and pp = 1, Figure 3).

**Figure 5:**
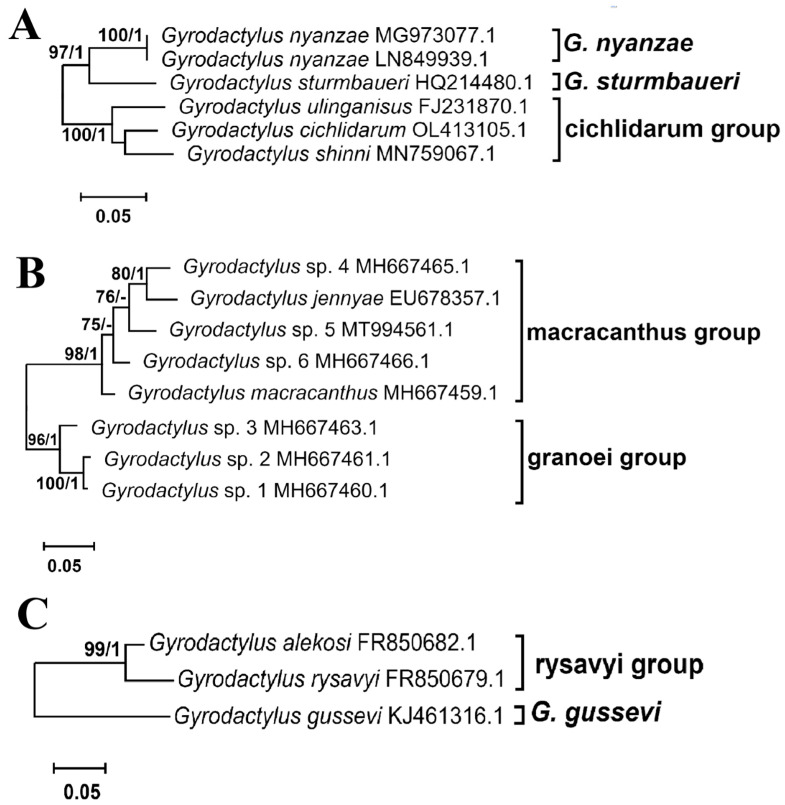
Expanded subtrees of *Cichlidarus* gen. nov. (**A**), *Macracanthus* gen. nov. (**B**), and *Rysavyius* gen. nov. (**C**) lineages from phylogenetic relationships inferred using ITS rDNA markers (Figure 3). Node numbers represent the ML bootstrap (bp) and BI posterior probability (pp) values. Values below bp = 75 and pp = 0.95 are not shown. Substitution models used in both analyses: ITS1—TVM+F+G4, 5.8S—SYM+G4, ITS2—TVM+F+G4.

Figure 5 illustrates the expanded subtrees of the three proposed novel genera, each containing multiple smaller species group. The new genus, *Cichlidarus* gen. nov. (Figure 5A), is comprised of three lineages, two singletons, and a well-supported species group: the cichlidarum group with bp = 100 and pp = 1. The two singletons are *Gyrodactylus sturmbaueri* Vanhove, Snoeks, Volckaert et Huyse, 2011 and *Gyrodactylus nyanzae* Paperna, 1973, which are sister species with a bootstrap value of 97 and a posterior probability of 1. The genus *Macracanthus* gen. nov. (Figure 5B) is a new addition to the classification and comprises two well-supported species groups: the macracanthus group, with bp = 98 and pp = 1, and the granoei group, with bp = 96 and pp = 1. Although the second group consists of three sequences from previously undescribed species, we have chosen to name them after *Gyrodactylus granoei* You, Guo, King et Cone, 2010 (HM185817.1). Although this reference sequence is incomplete and was thus excluded from the analyses, it was identified as the closest sequence to the undescribed species through BLAST analysis, with a query cover of 94% and a percent identity of 96%. The final proposed addition to the classification, *Rysavyius* gen. nov. (Figure 5C), comprises two distinct groups: the rysavyi group, which is well supported, with a bootstrap value of 99 and a posterior probability of 1; and a single sequence, KJ461316.1, which belongs to *Gyrodactylus gussevi* Dubey, Gupta et Agarwal, 1990.

### 3.3. Description of New Genera

Platyhelminthes Monot, 1876;
Monopisthocotyla (Odhner, 1912);
Gyrodactylidea Bykhovsky, 1937;
Gyrodactylidae (Beneden et Hesse, 1864)

#### 3.3.1. Cichlidarus gen. nov.

*Cichlidarus* gen. nov. Figures 1–3 and Table 1 in [89].

Type species: *Gyrodactylus cichlidarum* Paperna, 1968.
Current name of type species: *Cichlidarus* gen. nov. *cichlidarum* (Paperna, 1968).

Other species: *G. nyanzae* Paperna, 1973;
*G. shinni* Garcia-Vasques, Pinacho–Pinacho, Guzman–Valdivieso, Colixto–Rojas et Rubio–Gody, 2021;
*G. sturmbaueri* Vanhove, Snoeks, Volckaert et Huyse, 2011;
*Gyrodactylus ulinganisus* Garcia–Vasquez, Hansen, Christison, Bron et Shinn, 2011.
Current names of other species: *Cichlidarus* gen. nov. *nyaznaze* (Paperna, 1973);
*Cichlidarus* gen. nov. *shinni* (Garcia-Vasques, Pinacho–Pinacho, Guzman–Valdivieso, Colixto–Rojas et Rubio–Gody, 2021);
*Cichlidarus* gen. nov. *sturmbaueri* (Vanhove, Snoeks, Volckaert et Huyse, 2011);
*Cichlidarus* gen. nov. *ulinganisus* (Garcia–Vasquez, Hansen, Christison, Bron et Shinn, 2011).

Hologenotype for type species: ITS rDNA (ITS1–5.8S–ITS2 rDNA) sequences:
*Cichlidarus* gen. nov. *cichlidarum* OL413105.1.
Hologenotypes for other species:
*Cichlidarus* gen. nov. *nyanzae* MG973077.1;
*C.* gen. nov. *shinni* MN759067.1;
*C.* gen. nov. *sturmbaueri* HQ214480.1;
*C.* gen. nov. *ulinganisus* FJ231870.1;
  Cichlidarus gen. nov. sp. MN759067.

Type host: *Sarotherodon galilaeus* (L.) (Cichliformes: Cichlidae) [90].

Additional hosts: *Coptodon zillii* (Gervais) (Cichliformes: Cichlidae) [90];
*Haplochromis flaviijosephi* (Lortet) (Cichliformes: Cichlidae) [91];
*Hemichromis fasciatus* Peters (Cichliformes: Cichlidae) [90];
*Oreochromis niloticus* (L.) (Cichliformes: Cichlidae) [92];
*Oreochromis aureus* Steindachner (Cichliformes: Cichlidae) [91];
*Poeciliopsis gracilis* (Heckel) (Cyprinodontiformes: Poeciliidae) [93];
*Poecilia mexicana* Steindachner (Cyprinodontiformes: Poeciliidae) [93];
*Pseudoxiphophorus bimaculatus* (Heckel) (Cyprinodontiformes: Poeciliidae) [93];
*Rubricatochromis bimaculatus* (Gill) (Cichliformes: Cichlidae) [90];
*S. galilaeus galilaeus* (L.) (Cichliformes: Cichlidae) [91];
*Sarotherodon melanotheron heudelotii* (Duméril) (Cichliformes: Cichlidae) [91];
*Tilapia guineensis* (Günther) (Cichliformes: Cichlidae) [91];
*Tilapia zillii* (*Gervais*) (Cichliformes: Cichlidae) [91];
*Tristamella simonis simonis* (Günther) (Cichliformes: Cichlidae) [91].
Site on hosts: skin, fins, and gills.

Type locality: Accra plains and Akuse lagoon, Lower Volta, Ghana.

Type material: Holotype 35584, MRAC, Vouchers MRAC 37,560–37,562, Vouchers 2004.12.8.9–11 Natural History Museum, London, Vouchers M-406, Institute of Parasitology, Academy of Sciences of the Czech Republic, České Budějovice.

ZooBank registration: The Life Science Identifier (LSID) for *Gyrodactylus cichlidarum* Paperna, 1968 is urn:lsid:zoobank.org:act:18730161-3470-4E74-BEC4-29D3F526E1DA; the LSID for *Cichlidarus* gen. nov. is urn:lsid:zoobank.org:act:A90817FF-7DAA-4F9B-ABD6-5781883DC83D.

Etymology: The *Cichlidarus* gen. nov. name is derived from the species that is typical of the genus and was the first to be described.

Diagnosis: Hologenotype ITS rDNA (OL413105.1) 788 bp: ITS1 complete TTAAATT to AATTATA 343 bp, 5.8S complete CAACTCT to GTCGGCT 157 bp, ITS2 complete TTAACCT to TACTATT 289 bp. Opisthaptor typical for *Gyrodactylidae*, with 16 marginal hooks and a pair of anchors with ventral and dorsal bars. The ventral bar membrane is approximately square in shape with medial, spatulate ridges and with characteristic crescent-shaped depressions. Male copulatory organ (MCO): globular, with a single prominent apical spine and a single row of spikes, one set of robust “terminal” spikes, and two pairs of increasingly gracile “sub-terminal” and “medial” spikes. Excretory bladders present.

Annotations for other species’ ITS rDNA:
(MG973077.1), 801 bp—ITS1 complete TTAAATT to AATTATA 342 bp, 5.8S complete CAACTCT to GTCGGCT 157 bp, ITS2 TTTACCT to ATTTATT 302 bp;
(LN849939.1), not determined—ITS1 incomplete to TAATTATA, 5.8S complete CAACTCT to GTCGGCT 157 bp, ITS2 TTTACCT to incomplete;
(MN759067.1), 802 bp—ITS1 complete TTAAATT to AATAATA 342 bp, 5.8S complete CAACTCT to GTCGGCT 157 bp, ITS2 complete TTAACCT to CTTTAGT 303 bp;
(HQ214480.1), 799 bp—ITS1 complete TTAAATT to AATTATA 339 bp, 5.8S complete CAACTCT to GTCGGCT 157 bp, ITS2 complete ITS2 complete TTAATCT to AACTATT 303 bp;
(FJ231870.1) 802 bp—ITS1 complete TTAAATT to AATTATA 342bp, 5.8S complete CAACTCT to GTCGGCT 157 bp, ITS2 complete TTAACCT to CATTATT 303.

Remark: Detailed type species redescription is presented in [90]. Local phylogenies presented in [61,94] demonstrated that valid names for *Gyrodactylus chitandiri* Zahradničková, Barson, Luus-Powell et Přikrylová, 2016, *Gyrodactylus ergensi* Přikrylová, Matĕjusová, Musilová et Gelnar, 2009, *Gyrodactylus malalai* Přikrylová, Blažek et Gelnar, 2012, *Gyrodactylus occupatus* Zahradničková, Barson, Luus-Powell et Přikrylová, 2016, and *Gyrodactylus parisellei* Zahradnıčková, Barson, Luus-Powell et Přikrylová, 2016 should be changed into *Cichlidarus* gen. nov. *chitandiri* Zahradničková, Barson, Luus-Powell et Přikrylová, 2016, *Cichlidarus* gen. nov. *ergensi* Přikrylová, Matĕjusová, Musilová et Gelnar, 2009, *Cichlidarus* gen. nov. *malalai* Přikrylová, Blažek et Gelnar, 2012, *Cichlidarus* gen. nov. *occupatus* Zahradničková, Barson, Luus-Powell et Přikrylová, 2016, and *Cichlidarus* gen. nov. *parisellei* Zahradnıčková, Barson, Luus-Powell et Přikrylová, 2016, respectively. The ITS sequences of the above species were incomplete and therefore excluded from the analyses, but rDNA of *C.* gen. nov. *chitandiri* formed the sturmbaueri group with the singleton. *C.* gen. nov. *ergensi* and *C.* gen. nov. *malalai* formed the nyanzae group, and *C.* gen. nov. *occupatus* and *C.* gen. nov. *parisellei* joined the cichlidarum group.

#### 3.3.2. Iraquemembranatus gen. nov.

*Iraquemembranatus* gen. nov. Figure 10, Table 3, and Figure S5C in [68].

Type and the only species: *G. iraqemembranatus* Rahmouni, 2024 [68].
Current name: *Iraqemembranatus iraqemembranatus* (Rahmouni, 2024).

Hologenotype: ITS rDNA (ITS1-5.8S-ITS2 rDNA) sequence: *I. iraqemembranatus* OR773087.1.

Type host: *Paracapoeta trutta* (Heckel, 1843) (Cyprinoidei: Cyprinidae).
Additional hosts: *Alburnus sellal* Heckel, 1843 (Cyprinoidei: Leuciscidae); *Barbus lacerta* Heckel, 1843 (Cyprinoidei: Cyprinidae).
Site on the host: gill filaments for *P. trutta* and *B. lacerta*; fins for *A. sellal.*

Type locality: Kani Shok, tributary of the Tabin River, Sulaymaniyah Province, Iraq.

Type material: holotype and six paratypes (IPCAS M-784/1-3).

ZooBank registration: the Life Science Identifier (LSID) for *G. iraqemembranatus* Rahmouni, 2024 is urn:lsid:zoobank.org:act: B4738C07-9748-4217-80C0-D5510AC31E4F; the LSID for *Iraqemembranatus* gen. nov. is urn:lsid:zoobank.org:act:35264468-9620-45E3-9B70-9237899E06F8.

Etymology: the name derived from the original name *G. iraqemembranatus*.

Diagnosis: Hologenotype ITS rDNA (OR773087.1) 834 bp: ITS1 complete TGTATTG to TAATTTT 345 bp, 5.8S complete CAACTCC to GTCGGCT 157 bp, ITS2 complete TTTACCT to TTAGCCT 332 bp. Opisthaptor typical for *Gyrodactylidae*, with 16 marginal hooks and a pair of anchors with ventral and dorsal bars. The ventral bar is lacking bilateral processes and membrane. Male copulatory organ (MCO) with single prominent apical spine and a single row of spikes. Excretory bladders not investigated.

Remark: Detailed species description in [68]. *G. emembranatus* Malmberg, 1970 is another species with a ventral bar without bilateral processes and a membrane. Its phylogenetic position is uncertain. It is a marine species from *Gadus morhua* L. collected in the Norwegian Sea near Tromsø, classified by Malmberg [69] to the subgenus *G*. (*Metanephrotus*). The ITS rDNA sequence JF836148.1 [16] is from worms collected on *G. morhua* in the Atlantic Ocean near Nova Scotia. The sequence is incomplete and, therefore, was not used in the present analysis. Its phylogenetic position, inferred from partial 18S rDNA sequences, groups it with *Gyrocerviceanseris passamaquoddyensis* Cone, Abbott, Gilmore et Burt, 2010 (ITS1 rDNA not available either). When its ITS2 rDNA is blasted against the nucleotide database, it is grouped within the subgenus *G*. (*Gyrodactylus*).

#### 3.3.3. Macracanthus gen. nov.

*Macracanthus* gen. nov. (Plate 1: Figures 5 and 7 in [95], Figures 1 and 2 in [96], and Species B #2 FR15 30.4 in [67]).

Type species: *Gyrodactylus macracanthus* Hukuda, 1940.
Current name of type species: *Macracanthus* gen. nov. *macracanthus* (Hukuda, 1940).

Other species: *G. jennyae* Paetow, Cone, Huyse, McLaughlinand et Marcogliese, 2009;
*G. granoei* You, Guo, King et Cone, 2010.
Current names of other species: *Macracanthus* gen. nov. *jennyae* (Paetow, Cone, Huyse, McLaughlin et Marcogliese, 2009);
*Macracanthus* gen. nov. *granoei* (You, Guo, King et Cone, 2010).
Hologenotype: ITS rDNA (ITS1-5.8S-ITS2 rDNA) sequences: *Macracanthus* gen. nov. *macracanthus* MH667459.1.

Hologenotypes for other species:
*M.* gen. nov. *jennyae* EU678357.1;
*Macracanthus* gen. nov. sp. 1 MH667460.1.
*Macracanthus* gen. nov. sp. 2 MH667461.1;
*Macracanthus* gen. nov. sp. 3 MH667463.1;
*Macracanthus* gen. nov. sp. 4 MH667465.1;
*Macracanthus* gen. nov. sp. 5 MT994561.1;
*Macracanthus* gen. nov. sp. 6 MH667466.1;

Type host: *Misgurnus anguillicaudatus* (Cantor) (Cypriniformes: Cobitidae).
Additional hosts: not known.
Site on hosts: skin, fins, gills.

Type locality: Seoul, South Korea.

Type material: N/A.

ZooBank registration: the Life Science Identifier (LSID) for *G. macracanthus* Hukuda, 1940 is urn:lsid:zoobank.org:act:9B9904AC-684E-4095-8765-6FEA80C57B6A; the LSID for *Macracanthus* gen. nov. is urn:lsid:zoobank.org:act:FA3B583C-9FF5-42B9-BFE5-2D766A545BCC.

Etymology: The *Macracanthus* gen. nov. name is derived from the species typical of the genus, which was also the first to be described.

Diagnosis: Hologenetype ITS rDNA (MH667459.1) 838 bp): ITS1 complete TGTATTT to ATATGTA 359 bp, 5.8S complete CAACTCC to GTCGGCT 157 bp and ITS2 complete TTTACCT to ATTACTT 322 bp. Opisthaptor typical for *Gyrodactylidae*, with 16 marginal hooks and a pair of anchors with ventral and dorsal bars. Male copulatory organ (MCO) is globular. A pair of large triangular excretory bladders is present.
  Annotations for other species’ ITS rDNA:
  (EU678357.1)—855 bp—ITS1 complete CGTATTT to AATTATA 379 bp, 5.8S complete CAACTCC to GTCGGCT 157 bp, ITS2 complete TTTACCT to TTACTTG 319 bp;
  (MH667460.1)—842 bp, ITS1 complete TGTATTG to ATTTGTA 366 bp, 5.8S complete CAACTCC to GTCGGCT 157 bp, ITS2 complete TTTACCT to TTTGCCT 319 bp;
  (MH667461.1)—842 bp, ITS1 complete TGTATTG to ATTTGTA 366 bp, 5.8S complete CAACTCC to GTCGGCT 157 bp, ITS2 complete TTTACCT to TTTGCCT 319bp;
  (MH667463.1)—845 bp, ITS1 complete TGTATTG to ATTTGTA 368 bp, 5.8S complete CAACTCC to GTCGGCT 157 bp, ITS2 complete TTTACCT to TTAGCCT 320 bp;
  (MH667465.1)—840 bp, ITS1 complete CGTATTT to ATTTGTA 371 bp, 5.8S complete CAACTCC to GTCGGCT 157 bp, ITS2 complete TTTACCT to TTTCTAG 312 bp;
  (MT994561.1)—841 bp, ITS1 complete CGTATTT to ATTTGTA 365 bp, 5.8S complete CAACTCC to GTCGGCT 157 bp, ITS2 complete TTTACCT to TTATTTG 319 bp;
  (MH667466.1)—830 bp, ITS1 complete CGTATTT to ATTTGTA 354 bp, 5.8S complete CAACTCC to GTCGGCT 157 bp, ITS2 complete TTTACCT to TTACTTG 319 bp.

Remark: Detailed species description in [95,96]. In the local phylogeny presented in [67], *Gyrodactylus granoei* You, Guo, King et David Cone, 2010 is included. The ITS rDNA sequence of the species HM185817.1 is incomplete; therefore, it was not included in the analysis. However, the species belongs to *Macracantsus* gen. nov., so its name should be changed to *Macracantsus granoei* (You, Guo, King et David Cone, 2010).

#### 3.3.4. Rysavyius gen. nov.

*Rysavyius* gen. nov. (Figures 2 (j,k), 3 (a-c), Table 2 in [97]).

Type species: *Gyrodactylus rysavyi* Ergens, 1973.
Current name of type species: *Rysavyius* gen. nov. *rysavyi* (Ergens, 1973).

Other species: *G. alekosi* Přikrylová, Blažek et Vanhove, 2012;
*G. gussevi* Dubey, Gupta et Agarwal, 1990.
Current names of other species: *Rysavyius* gen. nov. *alekosi* (Přikrylová, Blažek et Vanhove, 2012);
*Rysavyius* gen. nov. *gussevi* (Dubey, Gupta et Agarwal,1990).

Hologenotypes: ITS rDNA (ITS1-5.8S-ITS2 rDNA) sequences: *Rysavyius* gen. nov. *rysavyi* FR850679.1;

Hologenotypes for other species:
*R.* gen. nov. *alekosi* FR850682.1;
*R.* gen. nov. *gussevi* KJ461316.1.

Type hosts: *Clarias gariepinus* (Burchell) (Siluriformes: Clariidae) [98].
Additional hosts: *C. anguillaris* (L., 1758) (Siluriformes: Clariidae) [97].
Site on hosts: fins and skin.

Type locality: River Nile and Abu Sarda, Egypt.

Type material: N/A.

ZooBank registration: the Life Science Identifier (LSID) for *G. rysavyi* Ergens, 1973 is urn:lsid:zoobank.org:act:68513FE2-E78D-40C1-9DD6-356A267C0491; the LSID for *Rysavyius* gen. nov. is urn:lsid:zoobank.org:act:6700E94B-7960-4848-9154-5A8E866B23B8.

Etymology: the name *Rysavyius* gen. nov. is derived from the species that is typical of the genus.

Diagnosis: Hologenetype ITS rDNA (FR850679.1) length not determined: ITS1 5′ end unreliable to ATTTGTA, 5.8S complete CAACTCC to GTCGGCT 157 bp, and ITS2 complete TTAACCT to TAAGCCT 384 bp. Opisthaptor typical for Gyrodactylidae, with 16 marginal hooks and a pair of anchors with ventral and dorsal bars. Anchors have a characteristic flattened area on the inner part of the root. Sickles have broad shafts and point downward, extending beyond the toe. Male copulatory organ (MCO) globular, with one large hook and eleven thin, small spikes in a single row. A pair of excretory bladders is present.
Annotation for other species’ ITS rDNA:
(FR850682.1)—not determined, ITS1 5′ end unreliable to ATTTGTA, 5.8S complete CAACTCC to GTCGGCT 157? bp and ITS2 complete TTAACCT to TAAGCCT 384 bp;
(KJ461316.1)—908 bp, ITS1 complete CGTATTG to ATTTGTA 367 bp, 5.8S complete CAACTCC to GTCGGCT 157 bp and ITS2 complete TTTACCT to AAACCTT 384 bp.

Remark: Detailed species description in [97]. An additional three species are present in the local phylogeny [97]—*Gyrodactylus nigritae* Přikrylová, Blažekand et Vanhove, 2012, *Gyrodactylus synodonti* Přikrylová, Blažekand et Vanhove, 2012, and Gyrodactylus sp. (FR850688). Their ITS rDNA sequences were incomplete; therefore, they were not included in the analysis. However, they belong to the new genus *Rysavyius* gen. nov., so their names should be changed to Rysavyius gen. nov. *nigritae* (Přikrylová, Blažek et Vanhove, 2012), *R.* gen. nov. *nigritae* (Přikrylová, Blažek et Vanhove, 2012) and *Rysavyius* gen. nov. sp., respectively.

## 4. Discussion

While the gyrodactylids’ complete rRNA gene operon is well known [99], and several studies have been conducted on it (IGS rDNA [100,101], 18S rDNA [8,11,13,14,16,17,18,23,26,54,55,59,102,103,104], 28S rDNA [81,105,106,107,108,109,110]), the most commonly used marker in the field is the internal transcribed spacer ribosomal DNA (ITS rDNA). The majority of contemporary papers describing new species or genotyping known species make use of sequences of the ITS rDNA marker [11,24,25,26,68,79,80,94,102,103,104,108,110,111,112,113,114,115,116,117,118,119,120,121,122,123,124,125]. Unfortunately, not all of these sequences were evaluated with regards to the presence of the expected external ends, which are highly conserved at the 3′ and 5′ ends of the 18S and 28S rDNA, respectively, and may indicate good sequence quality. Furthermore, the 157 bp length of the 5.8S rRNA gene is identical within Gyrodactylidae species and conserved across closely related groups, but this is also overlooked, judging by the presence of shorter or longer sequences in public databases. The lack of fundamental checks renders the alignment process more challenging and less reliable. While this issue can be partially addressed through the continued development of bioinformatics tools, the fundamental problem persists. One of the tools that facilitates significant sequence alignment and allows for the switching of sequences is MUSCLE v5.1 [81]. Phylogenetic analyses may be affected by such errors, leading to potential erroneous conclusions, and it is therefore preferable to remove such sequences from datasets.

The identification of species (and higher taxa) within the Gyrodactylidae is further hindered by the scarcity of diagnostic morphological characters. Current distinctions are based on subtle variations in haptor morphology (such as the shape and size of anchors, the presence of dorsal and ventral bars, and the presence of marginal hooks), pharynx morphology (which can have either short or long processes), MCO morphology (including the presence and arrangement of cyrrus small spikes), and the type of excretory system [69]. The latter can only be studied in live specimens, which is extremely difficult in remote areas with limited access to specialized equipment, where the vast majority of gyrodactylids usually live. These diagnostic taxonomic characters of the excretory system have attracted the attention of two researchers since the mid-1950s: Malmberg [69] and Gläser [78]. In the absence of an adequate number of morphological characters, molecular markers represent a viable alternative. At present, the ITS rDNA sequences represent the optimal choice. The objective of this project was to investigate the phylogenetic relationships of the most primitive *Gyrodactylus* species in the context of other primitive taxa within the Gyrodactylidae family. This was done in order to evaluate the biological significance of the obtained groupings. Malmberg [69] diagnosed the protonephridial system in only approximately 75 *Gyrodactylus* species. However, he also classified more than 200 species that had been described up to 1969. This indicates that, for over half of the species, other morphological traits were used for diagnostic purposes. In this study, we propose an alternative approach based on genetic relationships derived from nuclear DNA. We postulate that new, well-supported monophyletic species groupings with unique biological characteristics that are genetically distinct should be classified as new genera of the family. This approach should also involve a more detailed comparison of the morphological similarity of other biological characteristics, which would prove beneficial for the correct classification of the estimated 20,000 species within the Gyrodactylidae family [6]. This suggested approach is still clearly hampered by the use of incomplete species lists, leaving an open space for the discovery of further gyrodactylid genera as the list of species included is supplemented. Regardless, we suggest that the catch-all genus *Gyrodactylus* should then later be divided into multiple monophyletic groups that are consistent with the rest of the family’s genera. The first such group (*G*. (*Gyrodactylus*)), consisting of the most primitive species, has been presented in this paper. The initial phylogenies of the studied diversity, based on ITS rDNA [12,18,52,67,68,69], and the most comprehensive one, investigating *Gyrodactylus* species in Mongolia [112], have already been presented. It is worth noting that a review of the morphological characteristics of the species groupings observed in the phylogenetic analysis presented here should be required in order to evaluate their status.

A discussion of the phylogenetic position of the previously described genera used in the present phylogeny is unnecessary. Meanwhile, the ITS rDNA does not provide sufficient evidence to group existing subgenera (with the exception of *Gyrodactyloides* and *Ieredactylus*), because it is clear that they are genetically distinct. Similar outcomes were demonstrated by ML and BI phylogenies derived from combined sequences of V4 and ITS rDNA [103]. These genera also exhibit markedly disparate morphologies relative to *Gyrodactylus*. For example, *Ieredactylus* has a ventral bar membrane that is divided into two parts, along with triangular hamulus accessory pieces associated with the roots [28]. These characteristics are not observed in any currently known genera of the family Gyrodactylidae. In contrast, *Diplogyrodactylus* is characterized by the absence of a dorsal bar in its haptor, the presence of two distinct types of marginal hooks, and a tubular MCO. Furthermore, it possesses additional elements, namely, a pair of muscular adhesive disks lateral to the hamuli, which are distinctive of this species [27]. Furthermore, *Paragyrodactylus* exhibits a supplementary hard, helmet-like accessory part positioned over the hamulus roots within its haptor [20]. The principal hooks (anchors) of the *Macrogyrodactylus* genus are also worthy of note for their distinctive shape, which bears resemblance to that of bifurcated pincers. These morphological distinctions from the genus *Gyrodactylus* are readily apparent and are supported by a corresponding high degree of genetic divergence. The challenge appears when such pronounced morphological differences are not observed.

The lineage designated as the subgenus *G*. (*Gyrodactylus*) appears to be consistent with the taxon classified by Malmberg [69]. It should be noted that this classification also includes species that lack the protonephridial system under study, such as *Gyrodactylus alburnensis* Prost, 1972, *Gyrodactylus dulmaae* Ergens, 1970, *Gyrodactylus ellae* Rahmouni, Seifertová et Šimková, 2023, *Gyrodactylus huyseae* Rahmouni, Seifertová et Šimková, 2023, *Gyrodactylus kuchtai* Rahmouni, Seifertová and et Šimková, 2023, *Gyrodactylus neili* LeBlanc, Hansen, Burtet Cone, 2007, and *Gyrodactylus percotti* Ergens et Yukhimenko, 1973, as well as two undescribed species. Nevertheless, it is clear that they all belong to this lineage based on their genetic relationships.

In its initial classification, the subgenus was divided into two species groups: elegans and phoxini. The elegans group comprised three species: *Gyrodactylus elegans* Nordmann, 1832, *Gyrodactylus prostae* Ergens, 1964, and *G. laevis*. *G. elegans* and *G. prostae* Ergens, 1963 are sister species with a close evolutionary relationship. They exhibit consistent morphological characteristics that match the traits outlined by Malmberg [69]. The ventral bar is devoid of lateral processes and exhibits a relatively small, V-shaped membrane that is sometimes sharply pointed, as observed in *G. elegans*. In contrast, *G. laevis* is not included in the elegans group. It is a singleton, not affiliated with any other species group. It appears that the morphology of its ventral bar may be considered to be a plesiomorphic character. The ventral bar is devoid of lateral processes, and the membrane is relatively narrow with nearly parallel lateral edges, forming a “U” shape [126]. *G. alburnensis*, however, is included in this group, despite having been previously synonymized with *G. laevis* [127]. This is consistent with expectations, because its ventral bar morphology matches that of the elegans group [128]. The results of the phylogenetic analysis demonstrate that the synonymizing of this species, represented by the ITS rDNA sequence AY278032, was erroneous and resulted in the formation of a paraphyletic taxon. Therefore, the synonymizing should be abandoned. *Gyrodactylus decorus* Malmberg, 1957 is also included in the elegans group based on genetic data, contrary to Malmberg’s [69] classification, which instead places this species in the phoxini group. As observed through the use of microscope imaging techniques [129], the morphology of the ventral bar membrane exhibits a resemblance to the plesiomorphic shape with parallel edges. The genetic analysis also resulted in the inclusion of a newly described species, *G. huyseae*, within the elegans group. The ventral bar membrane of this species exhibits a notable resemblance to that of *G. decorus* and *G. alburnensis*, which possess a more plesiomorphic shape. It is thus proposed that the diagnosis of the elegans group be extended to include this haptor morphology. It is also noteworthy that *G. huyseae* is represented by two sequences (OR270000 and OR270001), as separated by genetic distance slightly higher than the 1.3% proposed for species-level differentiation by Ziętara and Lumme [73]. It can thus be posited with a high degree of probability that these sequences represent two distinct but closely related species. This hypothesis is also supported by the slight morphological differences observed between specimens collected from different hosts [102].

The second group of species described by Malmberg [69] within the subgenus *G*. (*Gyrodactylus*) was the phoxini group, which contains species possessing a ventral bar with small processes and a ventral bar membrane that is wider and more rounded near the end (spoon-shaped). The phoxini group, as defined by Malmberg [69], includes four species: *Gyrodactylus carassii* Malmberg, 1957, *Gyrodactylus phoxini* Malmberg, 1957, *Gyrodactylus magnificus* Malmberg, 1957, and *G. decorus*. The position of the last species, which was supported by phylogenetic DNA analysis based on ITS rDNA, has already been discussed. The remaining species form a monophyletic group that exhibits consistent morphological characteristics. The genetic analysis also suggests that *G. dulmaae* and *G. perccotti* should be included in the phoxini group. *G. dulmaae* is distinguished by having the narrowest ventral bar membrane among the phoxini group, which exhibits a less rounded end than other representatives of the group, thus resembling the plesiomorphic shape more closely. In contrast, the membrane of *G. perccotti* is broad at the base, narrows along its length, and does not exhibit a rounded end but, rather, a blunt truncation [130]. Additionally, it possesses small lateral processes [130,131]. It can thus be surmised that these additional morphological types represent a transitional state to more primitive forms. Nevertheless, the DNA analysis indicates that they belong to the phoxini group. In light of these findings, it is recommended that the diagnosis of this group be expanded. In conclusion, the morphology of the aforementioned species groups can be summarized as follows: The elegans group encompasses parasites that are characterized by a primitive U-shaped or narrow and sharply pointed ventral bar membrane. In contrast, the phoxini group typically exhibits a spoon-shaped membrane, although *G. dulmaae* and *G. perccotti* display a more primitive, less rounded structure. In contrast, *G. laevis* only possesses a membrane with a plesiomorphic U-shaped structure.

Two additional species groups were included based on the phylogeny of the internal transcribed spacer ribosomal DNA (ITS rDNA) sequence data: the sedelnikovi and neili groups. The first group, comprising three species (*Gyrodactylus barbatuli* Achmerov, 1952, *Gyrodactylus amurensis* Achmerov, 1952, and *Gyrodactylus sedelnikowi* Gvosdev, 1950), was not considered by Malmberg [69]. Malmberg was uncertain whether these species should be classified within the phoxini group of the *G*. (*Gyrodactylus*) subgenus. The phylogenetic analysis corroborated Malmberg’s reservations and excluded these species from the phoxini group. Therefore, it is necessary to ascertain the diagnostic characters of these species. A particularly distinctive feature of this group is the stocky, club-shaped anchor root, while the membrane of the ventral bar is U-shaped, and the marginal hooks exhibit a distinctive crescent-shaped foot.

The newly designated neili group comprises four species: *G. elle*, *G. neili*, *G. kuchtai*, and a yet-undescribed species, all indigenous to North America. Regarding their morphological characteristics, the shape of the ventral bar membrane is sub-rectangular and variable in width. This is particularly evident in *G. neili* [37], *G*. sp. 8 (see *Gyrodactylus* sp. “*C. neogaeus*” in [102]), and *G. kuchtai*. In *G. ellae*, the membrane exhibits a gentle folding, while the sickles of the marginal hooks are relatively long, with a notable foot and globose heel [37,102].

The present phylogenetic analysis lends support to the genus rank for the subgenus *G*. (*Gyrodactylus*) (Malmberg, 1970), as previously suggested by Gläser [70]. However, we believe that the further division of the genus *Gyrodactylus* (Nordmann, 1832) into more genera is still premature.

The four newly created genera in the family Gyrodactylidae—*Cichlidarus* gen. nov., *Iraqemembranatus* gen. nov., *Macracanthus* gen. nov., and *Rysavyius* gen. nov.—encompass all primitive gyrodactylid species described or identified within the genus *Gyrodactylus*, but which were not grouped into the most primitive subgenus *G*. (*Gyrodactylus*). The nomenclature of these novel subgenera was derived from the original names of the type species. The classification into species groups was based on the leading species, with preference given to those that were described first.

The genus *Cychlidarus* gen. nov., which exhibits a haptor and MCO morphology similar to that of the genus *Gyrodactylus*, encompasses gyrodactylids that are closely related to *Cychlidarus* gen. nov. *cichlidarum*. These species are known to infect fish belonging to the Cichlidae family, which are native to Africa. The genus is composed of three lineages, including two singletons and the cichlidarum group. The cichlidarum group exhibits remarkable consistency in its morphological characteristics. The marginal hook sickles can be distinguished by their shape, with a blunt foot at the anterior end and a round heel [49,61,89,94]. The group comprises three species: *Cichlidarus* gen. nov. *cichlidarum* (Paperna, 1968), *C.* gen. nov. *shinni* (Garcia-Vásquez, Pinacho-Pinach, Guzmán-Valdivieso, Calixto-Rojas et Rubio-Gody, 2011), and *C.* gen. nov. *ulinganisus* (Garcia-Vásquez, Hansen, Christison, Bron et Shinn, 2011). It appears that both singletons also represent two distinct species groups: the nyanzae and the sturmbaueri. The nyanzae group comprises gyrodactylids with marginal hooks that exhibit a teardrop-shaped foot, whereas the sturmbaueri group is characterized by marginal hooks with a more trapezoidal foot [52,61].

The genus *Iraqembranatus* gen. nov. exhibits a haptor and MCO morphology similar to that of the genus *Gyrodactylus*, but with a ventral bar devoid of lateral processes and a membrane. The marginal hook foot is characterized by a blunt toe that is slightly pointed outward. Excretory bladders were not investigated. A single species, *Iragemembranatus* gen. nov. *iragemembranatus* (Rahmouni, 2024), was described from an Iraqi cyprinid fish, *P. trutta*; however, it is not highly host-specific, and it has also been observed on other cyprinids, namely, *A. sellal* and *B. lacerta*. The high degree of morphological distinctiveness is reflected in the species’ phylogenetic position, as determined by ITS rDNA analysis [68]. Another species with a similar morphology is *Gyrodactylus emembranatus* Malmberg, 1970, which also exhibits other primitive characters.

The genus *Macracanthus* gen. nov. was formed to accommodate a group of East Asian freshwater gyrodactylids infecting cyprinids and loaches, which resemble the genus *Gyrodactylus* in both haptor and MCO morphology. These invasive species have been identified as a significant threat to the region’s biodiversity [67,132]. It is noteworthy that these primitive species appear to possess excretory bladders, which differentiate them from the subgenus *G*. (*Gyrodactylus*). The genus *Macracanthus* gen. nov. can be further divided into two species groups. One limitation of the current phylogenetic analysis is that one of the species groups is represented by three undescribed species, the morphological consistency of which requires further study. It should be noted, however, that in studies on invasive *Gyrodactylus* species in the USA, an incomplete sequence of *G. granoei* (HM185817) from *Cobitis granoei* was used. All three undescribed species were grouped in a single lineage together with the sequence HM185817; thus, we have designated this species group as the granoei group. The remaining sequences in the group originate from *Gyrodactys* spp. collected from *Pseudorasbora parva* in the remote eastern region of Russia [67]. The marginal hooks’ sickle shape is a distinctive feature of *G. granoei*, characterized by a prominent heel and a sickle point that extends beyond the foot’s toe [41]. The macracanthus group comprises five species, including three undescribed *Gyrodactylus* species. The most prominent species, *Macracanthus* sp. nov. *macracantus* (Hukuda, 1940), was initially described from *M. anguillicaudatus* in the Han River in Seoul. The specimen exhibited a pair of large bladders [95]. The species was subsequently redescribed, confirming the presence of all diagnostic characters [96]. Additionally, it exhibits the marginal hook sickle with a pronounced heel, although this is vertical in orientation, and the sickle point does not extend to the foot’s toe [41,96,132]. The second species in the group (*G. jennyae*) and species that have not yet been formally described [67] exhibit similar morphological characteristics, confirming the morphological consistency of the group. While all species within the macracanthus group parasitize *M. anguillicaudatus*, *G. jennyae* represents a significant pathogenic threat to American bullfrog (*Lithobates catesbeianus* Shaw, 1802) tadpoles [45], resulting from a globally invasive pet trade associated with a host switch [67].

The genus *Rysavyius* gen. nov. encompasses primitive gyrodactylids with haptor and MCO morphology analogous to that of the genus *Gyrodactylus*, yet with a protonephridial system comprising excretory bladders that are absent in the most primitive subgenus *G*. (*Gyrodactylus*). These parasites are known to infect freshwater catfishes from Africa (Clariidae and Mochokidae) and Asia (Heteropneustidae). In the phylogeny presented here, these organisms are represented by a singleton and a group of species referred to as the rysavyi group. The rysavyi group comprises two species: *R.* gen. nov. *rysavyi* (Ergens, 1973) from *Clarias gariepinus* (Burchell), and *R.* gen. nov. *alekosi* (Přikrylová, Blažek et Vanhove, 2011) from *Synodontis nigrita* Valenciennes. Both species exhibit a distinctive robust anchor with a flattened area on the internal part of the root, which narrows after its expanded juncture with the shaft. The sickles are characterized by a broad shaft that is directed downward, with points extended beyond the toe. The sickle foot is triangular in form [97]. The singleton *R.* gen. nov. *gussevi* (Dubey, Gupta etAgarwal, 1990) requires further investigation. The species was initially described as *G. gussevi*(Dubey, Gupta etAgarwal, 1990 from *Heteropneustes fossilis* (Bloch). However, this name was subsequently determined to be preoccupied by *G. gussevi* Ling, 1962, originally described from *Silurus soldatovi* Nikolskii et Soin. A third species, *G. gussevi* Najdenova, 1966, was described from *Gobius cobitis* Pallas and other gobiids, but the name was subsequently changed to *Gyrodactylus najdenovae* Malmberg, 1970. This name was later synonymized with *Gyrodactylus proterorhini* Ergens, 1967, but the priority rule was not clearly defined [31]. In GenBank, *G. gussevi* is treated as a junior synonym of *G. proterorhini*. However, this is only the case for *G. gussevi* Najdenova, 1966, not for the remaining two species. An ITS rDNA tag is absent from *G. gussevi* Ling, 1962, and the two remaining descriptions of *G. gussevi* are unclear [133,134]. Consequently, the status of both species remains undetermined, and redescriptions are necessary. The species represented by KJ461316 is grouped in the genus *Rysavyius* gen. nov. based on the ITS rDNA analysis, regardless of its final designation. The type host was determined as the catfish *H. fossilis*, and the type locality is one of the ponds in the Indian city of Raipur, situated within the catchment area of the Kharun River, a tributary of the Shivnath that ultimately flows to the Mahanadi and empties into the Bay of Bengal.

The substantial variation observed in ITS rDNA sequences appears to be a reliable indicator of the notorious morphological diversity among species within the family Gyrodactylidae. Conversely, closely related gyrodactylid species exhibit more similar morphological structures of their diagnostic characters compared to the genetic data. This pattern provides an opportunity to develop a classification system that can effectively delineate monophyletic groups for genera within the studied family, reflecting their evolutionary history. This will address the issue of the lack of sufficient diagnostic characters observed in parasitic taxa within the family Gyrodactylidae.

## Figures and Tables

**Table 1:** Mean likelihood and p-distances for the studied genera and subgenus *G*. (*Gyrodactylus*).

Genus	Mean Likelihood Distance	Mean p-Distance
*G*. (*Gyrodactylus*)	0.14	0.11
*Rysavyius* gen. nov.	0.39	0.20
*Cichlidarus* gen. nov.	0.09	0.08
*Macracanthus* gen. nov.	0.18	0.12
*Macrogyrodactylus*	0.10	0.08

The likelihood distances were estimated in IQTree2 [83] using the TVM+F+G4, SYM+G4, and TVM+F+G4 models for ITS1, 5.8S, and ITS2 regions respectively; p-distances were calculated in MEGA11 [82].

## Data Availability

The original contributions presented in the study are included in the article, further inquiries can be directed to the corresponding author/s.
